# Association between alanine aminotransferase and all‐cause mortality rate: Findings from a study on Japanese community‐dwelling individuals

**DOI:** 10.1002/jcla.24445

**Published:** 2022-04-18

**Authors:** Ryuichi Kawamoto, Asuka Kikuchi, Taichi Akase, Daisuke Ninomiya, Yoshio Tokumoto, Teru Kumagi

**Affiliations:** ^1^ Department of Community Medicine Ehime University Graduate School of Medicine Toon Japan; ^2^ Department of Internal Medicine Seiyo Municipal Nomura Hospital Seiyo‐city Japan

**Keywords:** alanine aminotransferase, all‐cause mortality, biomarker, cohort study, community‐dwelling individuals

## Abstract

**Background:**

This study examined the relationship between survival prognosis and alanine aminotransferase (ALT), a critical factor contributing to aging‐related health and mortality. The research is based on a follow‐up study with 6‐ and 10‐year intervals.

**Methods:**

The participants included 1,610 males (63 ± 14 years old) and 2,074 females (65 ± 12 years old) who were part of the Nomura cohort study conducted in 2002 (first cohort) and 2014 (second cohort). The multivariable‐adjusted hazard ratios (HRs) of death between the baseline health checkup and the end of the follow‐up periods were estimated using a Cox proportional hazards model, controlling for potential confounding factors.

**Results:**

The follow‐up survey revealed 180 male deaths (11.2% of male participants) and 146 female deaths (7.0% of female participants). The univariate Cox regression analysis showed a significant increase in the HRs of all‐cause mortality with decreasing ALT levels (*p* < 0.001). Furthermore, compared with individuals with ALT levels of 20–29 IU/L, the multivariable‐adjusted HRs (95% confidence interval) for all‐cause mortality were 2.73 (1.59–4.70) for those with ALT levels <10 IU/L, 1.45 (1.05–2.00) for those with ALT levels of 10–19 IU/L, and 1.63 (1.05–2.53) for those with ALT levels ≥30 IU/L.

**Conclusions:**

Our findings show that abnormally low ALT levels and high within the normal range were related to all‐cause mortality in Japan's community‐dwelling individuals. Especially, ALT activity may be an important biomarker for predicting the long‐term survival of older adults.

## Graphical abstract

Analysis of associations between alanine aminotransferase groups and all‐cause mortality during the follow‐up period using survival function. The *p*‐values were obtained through a log‐rank test of equality across various strata.

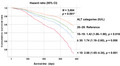

## 1 INTRODUCTION

Alanine aminotransferase (ALT) facilitates the transfer of amino groups as part of the reaction that forms pyruvate and glutamate, which are hepatic metabolite oxaloacetates. It is a reliable and sensitive biomarker of liver disease and can be detected using an inexpensive and routine biochemical assay.^1^ Elevated ALT levels can be a direct indicator of liver injury or inflammation, while ALT depletion can reflect reduced synthetic capacity. Both changes indicate hepatocellular dysfunction.

Laboratory findings regarding the relationship between ALT and health differ.^2^ Numerous researchers have suggested that serum ALT is a useful measure of overall health, major chronic diseases (for instance, liver disease, cancer, and diabetes),^3^ frailty,^4^ metabolic syndrome (MetS),^5^, ^6^ cardiovascular disease (CVD),^7^ and particularly obesity.^8^ Patients afflicted with these conditions are also at risk of developing nonalcoholic steatohepatitis.^9^

Research findings on the relationship between serum ALT levels and all‐cause mortality also remain inconsistent. For instance, one study reported a positive association between all‐cause mortality in participants without hepatitis virus infections and their levels of serum liver enzymes (including aspartate aminotransferase [AST], ALT, and gamma glutamyl transferase [GGT]), and similar associations were found for non‐liver disease mortality.^10^ Elevated serum ALT within the normal range is initially associated with lower mortality,^11^ although this effect is not observed at ≥17 U/L. Older adult populations tend to exhibit significantly less mortality with elevated ALT levels than do younger populations.^12^ When body mass index (BMI) is taken into account, the ability of serum ALT levels to predict prognosis is stronger for Japanese participants. More specifically, excess mortality was found to be associated with a combination of high serum ALT levels and below median BMI.^13^ On the other hand, significantly depleted ALT within normal range at the time of diagnosis is an independent risk factor contributing to increased mortality in elderly adults who have suffered an ischemic stroke^14^ and those with stable coronary heart disease.^15^, ^16^ Some researchers have reported a positive association between the ALT, even within normal range, and mortality from liver disease,^17^ although with inconsistent directions and magnitudes, which can be attributed to the varying ranges of ALT levels that were examined, inclusion of confounders, and differences in study populations.^11^

This study addresses these inconsistencies by investigating the potentially independent role of ALT in long‐term all‐cause mortality in community‐dwelling Japanese individuals based on sex, age, and BMI.

## 2 MATERIALS AND METHODS

### 2.1 Study design and participants

This prospective cohort analysis is part of the Nomura study,^18^ which was conducted in 2002 (first cohort) and 2014 (second cohort). Participants were primarily from rural areas in Ehime Prefecture and included those who had undergone community‐based annual health checkups. The first cohort comprised of 3,164 participants and the second consisted of 1,832 participants. All participants were aged between 22 and 95 years. Of these, 2,632 participants in the first cohort and 1,052 participants in the second cohort, not overlapping with the first cohort, underwent a similar baseline physical examinations and follow‐ups. Based on a self‐administered questionnaire, the study obtained data on the participants’ habits, medical history, current condition, and use of medication. Previous studies have presented flowcharts for the enrollment and exclusion of participants.^18^ Follow‐up surveys were conducted at 6‐ and 10‐year intervals for the first and second cohorts, respectively. Japan's Basic Resident Register was used to confirm participants’ survival status.

This study analyzed assessment data for the first and second cohorts (*N* = 3,684). The institutional review board (IRB) of Ehime University Hospital reviewed and approved the research (approval no. 1903018). Written informed consent was obtained from all participants.

### 2.2 Evaluation of risk factors

The participants’ weight and height were measured, and their weight (kg) was divided by their height (m^2^) to estimate BMI. Smoking status (in pack‐years) was calculated as the number of years an individual had been a smoker multiplied by the average number of packs per day. The categories of smoking status were nonsmokers, ex‐smokers, light smokers (<20 pack‐years), and heavy smokers (≥20 pack‐years). Daily alcohol intake was based on a unit of sake (22.9 g ethanol). The drinking categories were nondrinkers, occasional drinkers (<1 unit/day), light daily drinkers (1–2 units/day), and heavy daily drinkers (2–3 units/day). No participant consumed more than 3 units/day. Systolic blood pressure (SBP) and diastolic blood pressure (DBP) were measured using an automated sphygmomanometer. Participants were asked to rest for at least 5 min prior to the measurement and to remain in a seated position. An appropriately sized cuff was then placed on their right upper arm. Participants were also tested for triglycerides (TG), high‐density lipoprotein cholesterol (HDL‐C), low‐density lipoprotein cholesterol (LDL‐C), creatinine (Cr), serum uric acid (SUA), blood glucose (BG), GGT, ALT, and AST levels. They were required to undergo an overnight fast prior to these tests. The formula for the chronic kidney disease (CKD) epidemiology collaboration (CKD‐EPI) equation was modified using the Japanese coefficient to estimate the glomerular filtration ratio (eGFR) as follows.^19^ For males with Cr ≤0.9 mg/dl, this equation was used: 141 × (Cr/0.9)^−0.411^ × 0.993^age^ × 0.813; for males with Cr >0.9 mg/dl, the following was used: 141 × (Cr/0.9)^−1.209^ × 0.993^age^ × 0.813. For females with Cr ≤0.7 mg/dl, this equation was used: 144 × (Cr/0.7)^−0.329^ × 0.993^age^ × 0.813; and for females with Cr >0.7 mg/dl, the equation was as follows: 144 × (Cr/0.7)^−1.209^ × 0.993^age^ × 0.813. CVDs included ischemic heart disease, ischemic stroke, and peripheral vascular disease.

### 2.3 Statistical analysis

Statistical analyses were conducted using the program IBM SPSS Statistics (version 27.0; SPSS). Mean ± standard deviation (SD) is used to represent continuous variables. For non‐normal variables (TG, BG, GGT, ALT, and AST), the median and interquartile range are reported. For parameters with non‐normal distributions, all analyses employed log‐transformed values. Participants were divided into four groups based on their ALT levels (very low: <10 IU/L; low: 10–19 IU/L; medium: 20–29 IU/L; and high: ≥30 IU/L). Chi‐squared tests were conducted to compare categorical variables, and student's *t* tests were performed to compare normally distributed continuous variables. A univariable analysis based on the Cox proportional hazards model was performed for each baseline characteristic, and all confounding factors determined to be significant were incorporated as covariates. Next, the forced entry method was used to conduct a multivariable analysis that was based on the Cox proportional hazards model, with age as the primary time variable. Consistency in the observed association between ALT levels and all‐cause mortality was determined by performing subgroup analyses. Next, a likelihood ratio test was used to examine interactions between ALT groups and subgroup variables. The effect variable was assessed using an interaction test, which adjusted for all significant confounding variables (except the effect variable). All *p*‐values were two sided, and *p* < 0.05 was considered significant.

## 3 RESULTS

### 3.1 Baseline Characteristics of participants stratified by ALT categories

The sample comprised 3,684 participants, of whom 43.7% were male. The mean age was 64 ± 13 years. The median (interquartile range) follow‐up period was 3,160 (2,330–3,693) days. Follow‐up surveys confirmed that there were 326 (8.8%) deaths, of which 180 were male (11.2% of all males) and 146 were female (7.0% of all females). Table 1 presents the participants’ baseline characteristics stratified by ALT categories. Participants in the lowest and highest ALT categories were younger than those in other ALT categories. The prevalence of male, smoking habits, drinking habits, SBP, DBP, use of antihypertensive medication, use of TG, use of lipid‐lowering medication, BG, use of antidiabetic medication, SUA, use of SUA‐lowering medication, GGT, and AST levels increased significantly with increasing ALT category. However, HDL‐C levels decreased significantly with increasing ALT levels. This study found no difference between ALT categories in terms of history of CVD or LDL‐C.

**TABLE 1:** Baseline characteristics of participants stratified by alanine aminotransferase category

Characteristics *n* = 3,684	Alanine aminotransferase categories (IU/L)				*p*‐value *
	<10	10–19	20–29	≥30	
	*n* = 154	*n* = 2,088	*n* = 943	*n* = 499	
Gender (male), %	23.4	34.0	56.9	65.7	**<0.001**
Age (years)	60 ± 16	65 ± 12	64 ± 12	60 ± 13	**<0.001**
Body mass index (kg/m^2^)	21.8 ± 2.9	22.7 ± 3.0	23.8 ± 3.0	25.2 ± 3.6	**<0.001**
Smoking habits (never/past/light/heavy), %	55.2/31.8/9.7/3.2	58.1/24.3/11.9/5.7	44.9/27.9/15.0/12.3	36.5/25.9/19.2/18.4	**<0.001**
Drinking habits (never/occasional/light/heavy), %	77.3/20.8/0.6/1.3	72.3/21.7/2.3/3.8	59.5/28.8/3.3/8.4	51.9/37.5/4.0/6.6	**<0.001**
History of cardiovascular disease, %	10.4	8.0	8.1	7.4	0.697
Systolic blood pressure (mmHg)	132 ± 23	137 ± 22	139 ± 20	139 ± 19	**<0.001**
Diastolic blood pressure (mmHg)	76 ± 11	79 ± 11	81 ± 11	83 ± 11	**<0.001**
Use of antihypertensive medication, %	24.0	29.8	33.5	34.5	**0.016**
Triglycerides (mg/dl)	79 (61–114)	88 (67–119)	96 (72–138)	120 (85–173)	**<0.001**
HDL cholesterol (mg/dl)	64 ± 16	64 ± 16	62 ± 16	59 ± 16	**<0.001**
LDL cholesterol (mg/dl)	113 ± 31	119 ± 30	119 ± 31	117 ± 35	0.149
Use of lipid‐lowering medication, %	5.8	8.7	11.2	12.0	**0.014**
Blood glucose (mg/dl)	93 (86–102)	99 (90–114)	102 (92–117)	103 (93–118)	**<0.001**
Use of antidiabetic medication, %	4.5	7.6	10.8	12.0	**<0.001**
eGFR (ml/min/1.73 m^2^)	80.1 ± 18.7	76.8 ± 16.4	78.9 ± 15.6	81.1 ± 16.6	**<0.001**
Serum uric acid (mg/dl)	4.6 ± 1.3	4.9 ± 1.3	5.4 ± 1.4	5.9 ± 1.5	**<0.001**
Serum uric acid lowering medication, %	3.2	2.9	4.5	9.4	**<0.001**
Gamma glutamyl transferase (IU/L)	16 (14–22)	20 (15–28)	30 (22–49)	56 (33–109)	**<0.001**
Alanine transaminase (IU/L)	9 (8–9)	14 (12–17)	23 (21–26)	38 (33–50)	**<0.001**
Aspartate transaminase (IU/L)	16 (14–19)	21 (18–24)	26 (22–30)	34 (29–44)	**<0.001**

Note

Data are presented as mean ± standard deviation. Data for triglycerides, blood glucose, gamma glutamyl transferase, alanine transaminase, and aspartate transaminase were skewed and are thus presented as median (interquartile range) values and log‐transformed for analysis. *p‐values are from ANOVA tests for continuous variables or from χ^2^ tests for categorical variables. Significant values (*p* < 0.05) are presented in bold.

### 3.2 Kaplan–Meier survival curves for relationships between the four ALT categories and all‐cause mortality

**FIGURE 1:**
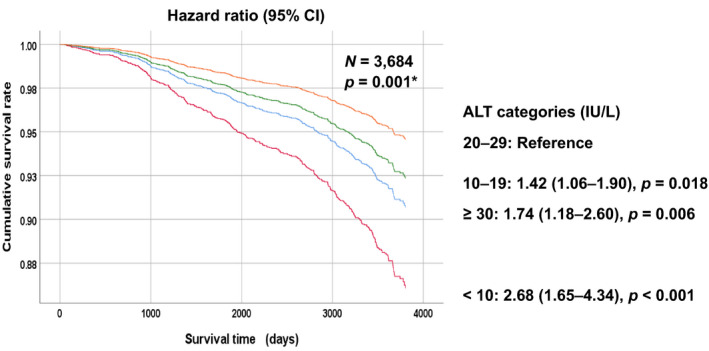
Analysis of associations between alanine aminotransferase groups and all‐cause mortality during the follow‐up period using survival function. ALT, alanine aminotransferase. ALT categories: Very low: <10 IU/L; low: 10–19 IU/L; medium: 20–29 IU/L; high: ≥30 IU/L). *Adjusted for gender and age. Log‐rank test. The *p*‐values were obtained through a log‐rank test of equality across various strata

Figure 1 presents adjusted survival curves for survival days and cumulative survival rates. The curves were plotted to identify patterns in the relationships between the four ALT categories and all‐cause mortality. The results indicate a significantly lower cumulative survival rate for individuals with ALT levels <10 IU/L (hazard ration [HR], 2.68; 95% confidence interval [CI], 1.65–4.34) and ≥30 IU/L (HR, 1.74; 95% CI, 1.18–2.60), compared with those with ALT levels of 20–29 IU/L.

### 3.3 Adjusted hazard ratios and 95% confidence intervals of baseline characteristics for all‐cause mortality

We identified both quantitative and categorical variables in the univariable and multivariable analyses (see Table 2 for the HRs and 95% CIs for the variables). The significant predictors of all‐cause mortality in the univariable models were gender, age, BMI, history of CVD, SBP, use of antihypertensive medication, LDL‐C, BG, use of antidiabetic medication, eGFR, SUA, use of SUA‐lowering medication, ALT, and AST. In the multivariable model, significant predictors were gender, age, BMI, history of CVD, SBP, LDL‐C, use of antidiabetic medication, SUA, ALT, and AST.

**TABLE 2:** Adjusted hazard ratios and 95% confidence intervals of baseline characteristics for all‐cause mortality

Characteristics *n* = 3,684	Univariable		Multivariablea^a^	
	HR (95% CI)	*p*‐Valu*e*	HR (95% CI)	*p*‐Value
Gender (men =1, women =2)	0.60 (0.49–0.75)	**<0.001**	0.73 (0.56–0.94)	**0.016**
Age (per 1 year increase)	1.10 (1.09–1.11)	**<0.001**	1.09 (1.07–1.11)	**<0.001**
Body mass index (per 1 kg increase)	0.92 (0.88–0.95)	**<0.001**	0.93 (0.89–0.97)	**0.001**
Smoking habits (never =1, past =2, light =3, heavy =4)	1.12 (0.96–1.30)	0.160	‐	
Drinking habits (never =1, occasional =2, light =3, heavy =4)	1.01 (0.90–1.12)	0.930	‐	
History of cardiovascular disease (no =0, yes =1)	2.43 (1.82–3.24)	**<0.001**	1.38 (1.02–1.85)	**0.035**
Systolic blood pressure (per 1 mmHg increase)	1.02 (1.01–1.02)	**<0.001**	1.01 (1.00–1.01)	**0.033**
Diastolic blood pressure (per 1 mmHg increase)	1.01 (1.00–1.02)	0.154	‐	
Use of antihypertensive medication (no =0, yes =1)	1.75 (1.41–2.19)	**<0.001**	1.04 (0.82–1.32)	0.771
Triglycerides (per 1 mg/dl increase)	0.82 (0.65–1.03)	0.088	‐	
HDL cholesterol (per 1 mg/dl increase)	1.00 (0.99–1,01)	0.845	‐	
LDL cholesterol (per 1 mg/dl increase)	0.99 (0.99–1.00)	**<0.001**	0.99 (0.99–1.00)	**0.001**
Lipid‐lowering medication (no =0, yes =1)	1.19 (0.82–1.73)	0.366	‐	
Blood glucose (per 1 mg/dl increase)	2.33 (1.46–3.73)	**<0.001**	0.85 (0.41–1.78)	0.667
Use of antidiabetic medication (no =0, yes =1)	1.94 (1.44–2.62)	**<0.001**	1.66 (1.10–2.49)	**0.015**
eGFR (per 1 ml/min/1.73 m^2^ increase)	0.98 (0.97–0.99)	**<0.001**	1.01 (1.00–1.01)	0.174
Serum uric acid (per 1 mg/dl increase)	1.11 (1.03–1.20)	**0.005**	1.11 (1.01–1.22)	**0.032**
Use of serum uric acid‐lowering medication (no =0, yes =1)	1.70 (1.11–2.63)	**0.016**	1.05 (0.66–1.67)	0.834
Gamma glutamyl transferase (per 1 IU/L)	1.05 (0.90–1.23)	0.500	‐	
Alanine transaminase (per 1 IU/L)	0.67 (0.52–0.86)	**0.001**	0.54 (0.35–0.85)	**0.007**
Aspartate transaminase (per 1 IU/L)	1.56 (1.14–2.13)	**0.005**	1.94 (1.08–3.49)	**0.027**

Note

Data for triglycerides, blood glucose, GGT, ALT, and AST were skewed and therefore log‐transformed for analysis. Significant values (*p* < 0.05) are presented in bold.

Abbreviations: CI, confidence interval; HR, hazard ratio.

^a^ Multivariate‐adjusted HR: adjusted for all confounding factors that were significant by a univariable analysis based on the Cox proportional hazards model.

### 3.4 Hazard ratios and 95% confidence intervals of baseline ALT categories for all‐cause mortality by age group

Table 3 shows that participants in the ALT categories <10 IU/L, 10–19 IU/L, and ≥30 IU/L had a higher risk of all‐cause mortality than those in the 20–29 IU/L category. The analysis was adjusted for gender, age, BMI, history of CVD, SBP, use of antihypertensive medication, LDL‐C, BG, use of antidiabetic medication, eGFR, SUA, use of SUA‐lowering medication, and AST. The results still showed a significant association between risk of all‐cause mortality and all ALT categories except 20–29 IU/L. When the participants were categorized by age (< 65 years and ≥65 years), this association was found only in the older age group.

**TABLE 3:** Hazard ratios and 95% confidence intervals of baseline alanine aminotransferase categories for all‐cause mortality by age group

ALT categories	Prevalence of death/total (%)	Nonadjusted HR (95% CI)	Gender and age‐adjusted HR (95% CI)	Multivariable‐adjusted HR (95% CI)^a^
Overall (*n* = 3,684)				
<10 IU/L	23/154 (14.9)	**2.19 (1.35–3.53)**	**2.68 (1.65–4.34)**	**2.73 (1.59–4.70)**
10–19 IU/L	201/2,088 (9.6)	**1.49 (1.12–1.99)**	**1.42 (1.06–1.90)**	**1.45 (1.05–2.00)**
20–29 IU/L	61/943 (6.5)	1.00	1.00	1.00
≥30 IU/L	41/499 (8.2)	1.25 (0.84–1.86)	**1.75 (1.17–2.60)**	**1.63 (1.05–2.53)**
*p* for trend	**0.001**	**0.005**	**<0.001**	**0.001**
<65 years of age (*n* = 1,571)				
<10 IU/L	3/83 (3.6)	1.18 (0.33–4.19)	2.25 (0.61–8.26)	2.47 (0.59–10.3)
10–19 IU/L	27/787 (3.4)	1.19 (0.60–2.34)	1.42 (0.72–2.84)	1.69 (0.78–3.63)
20–29 IU/L	12/422 (2.8)	1.00	1.00	1.00
≥30 IU/L	12/279 (4.3)	1.48 (0.67–3.30)	1.43 (0.64–3.20)	1.14 (0.45–2.85)
*p* for trend	0.781	0.815	0.592	0.526
≥65 years of age (*n* = 2,113)				
<10 IU/L	20/71 (28.2)	**2.96 (1.76–4.99)**	**2.62 (1.55–4.42)**	**2.62 (1.45–4.74)**
10–19 IU/L	174/1,301 (13.4)	**1.42 (1.03–1.94)**	**1.39 (1.00–1.91)**	1.40 (0.98–2.00)
20–29 IU/L	49/521 (9.4)	1.00	1.00	1.00
≥30 IU/L	29/220 (13.2)	1.45 (0.91–2.29)	**1.83 (1.16–2.90)**	**1.74 (1.05–2.88)**
*p* for trend	**<0.001**	**0.002**	**0.002**	**0.003**

Note

Significant values (*p* < 0.05) are presented in bold.

Abbreviations: ALT, alanine aminotransferase; CI, confidence interval; HR, hazard ratio.

^a^ Multivariate‐adjusted HR: adjusted for gender, age, body mass index, history of cardiovascular disease, systolic blood pressure, use of antihypertensive medication, LDL cholesterol, blood glucose, use of antidiabetic medication, eGFR, serum uric acid, use of serum uric acid‐lowering medication, and aspartate transaminase.

### 3.5 Hazard ratios and 95% confidence intervals of baseline ALT categories for all‐cause mortality by subanalysis

Table 4 stratifies participants by gender, age (<65 and ≥65 years), BMI (<25 kg/m^2^ and ≥25 kg/m^2^), history of CVD, and time until death (<1,095 days or ≥1,095 days). Similar to our previous results, a lower risk of all‐cause mortality was associated with higher ALT levels within the normal range. This association holds particularly for men, participants aged 65 years and above, and those with BMI <25 kg/m^2^, irrespective of history of CVD.

**TABLE 4:** Hazard ratios and 95% confidence intervals of baseline alanine aminotransferase categories for all‐cause mortality by subanalysis

Characteristics *n* = 3,684	Multivariable‐adjusted HR (95% CI)^a^ ALT categories				*p* for trend	*p* for interaction
	<10 IU/L	10–19 IU/L	20–29 IU/L	≥30 IU/L		
Gender						
Men (*n* = 1,610)	**2.88 (1.35–6.15)**	**1.60 (1.06–2.40)**	1.00	1.59 (0.93–2.73)	**0.017**	0.920
Women (*n* = 2,074)	2.22 (0.99–5.01)	1.17 (0.69–1.99)	1.00	1.69 (0.78–3.64)	0.083	
Age						
<65 years (*n* = 1,571)	2.47 (0.59–10.3)	1.69 (0.78–3.63)	1.00	1.14 (0.45–2.85)	0.526	0.875
≥65 years (*n* = 2,113)	**2.62 (1.45–4.74)**	1.40 (0.98–2.00)	1.00	**1.74 (1.05–2.88)**	**0.003**	
Body mass index						
<25 kg/m^2^ (*n* = 2,681)	**2.40 (1.34–4.30)**	1.26 (0.87–1.81)	1.00	**1.89 (1.12–3.17)**	**0.002**	0.339
≥25 kg/m^2^ (*n* = 1,003)	2.48 (0.30–20.5)	**2.15 (1.10–4.24)**	1.00	0.98 (0.41–2.35)	0.139	
History of cardiovascular disease						
No (*n* = 3,389)	**2.74 (1.50–5.01)**	**1.43 (1.01–2.04)**	1.00	1.35 (0.83–2.21)	**0.008**	0.154
Yes (*n* = 295)	2.31 (0.63–8.49)	1.63 (0.71–3.74)	1.00	**4.39 (1.47–13.0)**	**0.046**	
Time to death						
<1,095 days (*n* = 73)	Not examined					Not examined
≥1,095 days (*n* = 3,611)	**2.62 (1.41–4.90)**	**1.51 (1.05–2.18)**	1.00	**1.67 (1.01–2.76)**	**0.006**	

Note

Significant values (*p* < 0.05) are presented in bold.

Abbreviations: CI, confidence interval; HR, hazard ratio.

^a^ Multivariate‐adjusted HR: adjusted for gender, age, body mass index, history of cardiovascular disease, systolic blood pressure, use of antihypertensive medication, LDL cholesterol, blood glucose, use of antidiabetic medication, eGFR, serum uric acid, use of serum uric acid‐lowering medication, and aspartate transaminase.

## 4 DISCUSSION

The findings of this cohort study highlight ALT as an independent and significant predictor of all‐cause mortality in community‐dwelling individuals. After adjusting for confounders, the HR (95% CI) for all‐cause mortality was 2.73 (1.59–4.70) for those with ALT levels less than 10 IU/L, 1.45 (1.05–2.00) for those with ALT levels between 10 IU/L and 19 IU/L, and 1.63 (1.05–2.53) for those with ALT levels greater than or equal to 30 IU/L, when compared with individuals with ALT levels between 20 IU/L and 29 IU/L. All participants who died within 3 years of the follow‐up period were excluded, to address the issue of reverse causality; the effect of this on the results was minor. ALT levels were also significantly associated with participants who died at age 65 and above or who had a BMI of less than 25 kg/m^2^, irrespective of history of CVD. To the best of the authors’ knowledge, few studies have examined the relationship between ALT levels within the normal range and all‐cause mortality among community‐dwelling individuals in Japan.^10^, ^20^

Research findings on the relationship between ALT and all‐cause mortality are inconsistent, and this can be attributed to the influence of confounding variables.^21^ A study that looked at community residents in the United States found that high serum ALT levels reported in a routine medical care setting were associated with future mortality.^22^ Compared with participants who exhibited ALT concentrations of <20 IU/L, the adjusted relative risks (95% CI) of ALT concentrations of 20–29 IU/L and 30–39 IU/L were 2.9 (2.4–3.5) and 9.5 (7.9–11.5) for male participants and 3.8 (1.9–7.7) and 6.6 (1.5–25.6) for female participants.^17^ Similarly, the predictive value of serum ALT levels for prognosis was more evident for Japanese participants when we accounted for BMI. Thus, there was a significant association between excess mortality and the combination of high serum ALT levels and below median BMI.^20^ However, this study revealed a U‐shaped relationship between serum ALT concentration and increased risk of all‐cause mortality, and this finding is consistent with several existing studies.^2^, ^23^, ^24^, ^25^ Oh et al.^25^ found a linear relationship between serum ALT levels and all‐cause mortality in adults younger than 60 years, although they obtained a U‐shaped curve for adults aged 60 years and above. The National Health and Nutrition Examination Survey, which involved 15,028 adults, reported that multivariate‐adjusted mortality was higher for the lowest ALT decile (decile 1) (HR: 1.42; 95% CI: 1.24–1.63), decile 2 (HR: 1.27; 95% CI: 1.06–1.53), and decile 3 (HR: 1.25; 95% CI: 1.04–1.50). However, it was not significantly higher for decile 10 (HR: 1.21; 95% CI: 0.91–1.61) when compared with deciles 4–9.^24^ Research has shown an inverse and independent association between serum ALT levels and all‐cause mortality among older adult populations^12^ and in certain participants (for instance, those with CVD).^15^, ^16^ Thus, the present work is one of few observational studies that focus on subjects with ALT levels within the normal range.^27^

Understanding of the mechanisms underpinning increased all‐cause mortality in individuals with considerably low ALT levels remains insufficient. This has been speculatively attributed to the role of ALT in skeletal muscle and liver injury.^28^ ALT is clinically noteworthy when related activity is elevated in muscle or liver disease. This may be caused by the release of enzymes from damaged cells into the bloodstream. Thus, it is possible that individuals with liver disease and high ALT levels tend to die earlier. However, this raises the question of what mechanism contributes to a decline in ALT activity such that the risk of death increases when BMI <25 kg/m^2^ or during old age. Another possibility is that extremely low ALT levels may indicate frailty or sarcopenia.^24^, ^27^ Serum ALT levels in frail older adults are extremely low because of liver degeneration and reduced ALT production.^4^, ^29^ Finally, individuals who are overweight or obese tend to have fatty livers (for instance, nonalcoholic fatty liver disease), which are characterized by a moderate increase in ALT. Various studies have reported a paradoxical relationship between weight and survival in old age, where survival rates improve in overweight and even obese people.^30^, ^31^ This study showed an association between BMI and ALT, wherein participants with BMI <25 kg/m^2^ and higher ALT levels within the normal range tended to have a lower risk of all‐cause mortality. However, dysregulation of blood glucose, even in prediabetes, may be associated with the abnormal ALT,^32^ and even in prediabetes, it was associated with higher risk of all‐cause mortality.^33^ ALT elevation was associated with deaths from liver disease, but not from CVD, neoplasms, or diabetes.^34^ In this study, ALT levels within the normal range were significantly associated with all‐cause mortality, independently of gender, age, BMI, history of CVD, SBP, use of antihypertensive medication, LDL‐C, BG, use of antidiabetic medication, eGFR, SUA, use of SUA lowering medication, and AST.

This study provides key insights into Japan's rural population, although it is not free from limitations. Firstly, this study measured baseline characteristics and ALT levels during the initial visit in a cross‐sectional manner. However, ALT levels and some covariates tend to vary over time and may have changed during the long‐term follow‐up period. Thus, the relevance of this study may be underestimated, rather than overestimated, owing to nondiscriminatory misclassification bias. Second, the survey recorded all deaths (including those caused by internal and external factors) registered in Japan's Basic Resident Register. It is possible that people who relocated during the survey period were excluded. Third, the baseline included many confounding factors (for instance, medications, underlying diseases, and lifestyle modifications) that have been previously reported to be associated with death, although corrected with as many confounding factors as available on baseline physical examinations. Further research on the influence of factors that have not been evaluated here is thus warranted. Finally, the causal relationship between ALT levels and all‐cause mortality may have been underestimated here, given the relatively small number of participants and deaths.

In conclusion, this study observed that abnormally low ALT levels and high within the normal range were related to all‐cause mortality in Japan's community‐dwelling individuals. Finally, future large‐scale cohort studies with long‐term follow‐up periods are needed to examine the association between low ALT levels and mortality.

## CONFLICT OF INTEREST

The authors declare no competing interests.

## AUTHOR CONTRIBUTIONS

RK and AK participated in the study design, performed the statistical analyses, and drafted the manuscript. RK, AK, DN, YT, TA, and TK contributed to data acquisition and interpretation. RK and AK contributed to the conception and design of the statistical analyses. RK conceived the study, participated in its design, coordinated the writing of and helped draft the manuscript. All authors have read and approved the manuscript.

## Data Availability

The data that support the findings of this study are available on request from the corresponding author. The data are not publicly available due to privacy or ethical restrictions.
